# Vaccination against Atherosclerosis: Is It Real?

**DOI:** 10.3390/ijms23052417

**Published:** 2022-02-22

**Authors:** Anastasia V. Poznyak, Evgeny E. Bezsonov, Tatyana V. Popkova, Antonina V. Starodubova, Alexander N. Orekhov

**Affiliations:** 1 Institute for Atherosclerosis Research, Skolkovo Innovative Center, 121609 Moscow, Russia; 2 Laboratory of Cellular and Molecular Pathology of Cardiovascular System, Institute of Human Morphology, 3 Tsyurupa Street, 117418 Moscow, Russia; evgeny.bezsonov@gmail.com; 3 Laboratory of Angiopathology, Institute of General Pathology and Pathophysiology, 8 Baltiiskaya Street, 125315 Moscow, Russia; 4 Department of Biology and General Genetics, I.M. Sechenov First Moscow State Medical University (Sechenov University), 8 Izmailovsky Boulevard, 105043 Moscow, Russia; 5 V.A. Nasonova Institute of Rheumatology, 34A Kashirskoye Shosse, 115522 Moscow, Russia; popkovatv@mail.ru; 6 Federal Research Centre for Nutrition, Biotechnology and Food Safety, 2/14 Ustinsky Passage, 109240 Moscow, Russia; avs.ion@yandex.ru; 7 Medical Faculty, Pirogov Russian National Research Medical University, 1 Ostrovitianov Street, 117997 Moscow, Russia

**Keywords:** atherosclerosis, CVD, immunity, vaccination

## Abstract

Atherosclerosis has been known in medicine for several centuries. As early as 1755, the Swedish anatomist Albrecht von Haller used the term “atheroma” to describe vascular lesions. Atherosclerosis may originate from an unbalanced diet or bad habits, and is mainly found in developed countries. Clinical trials have been conducted to establish the causes of atherosclerosis, and also to develop treatments for this disease. However, prevention of the disease has always been better than treatment, so vaccination may be the key to saving thousands of lives. The creation of a vaccine may be directly related to the study of autoimmune processes occurring in the body, immunity. This review considers the issues related to the involvement of the immune response in the development of atherosclerotic lesions. Modern concepts of atherogenesis, immune inflammation in atherosclerosis, and potential vaccine targets are also discussed. There is a particular focus on experimental and clinical data supporting the development of immune therapies to reduce cardiovascular risk.

## 1. Introduction

**Scheme 1:**
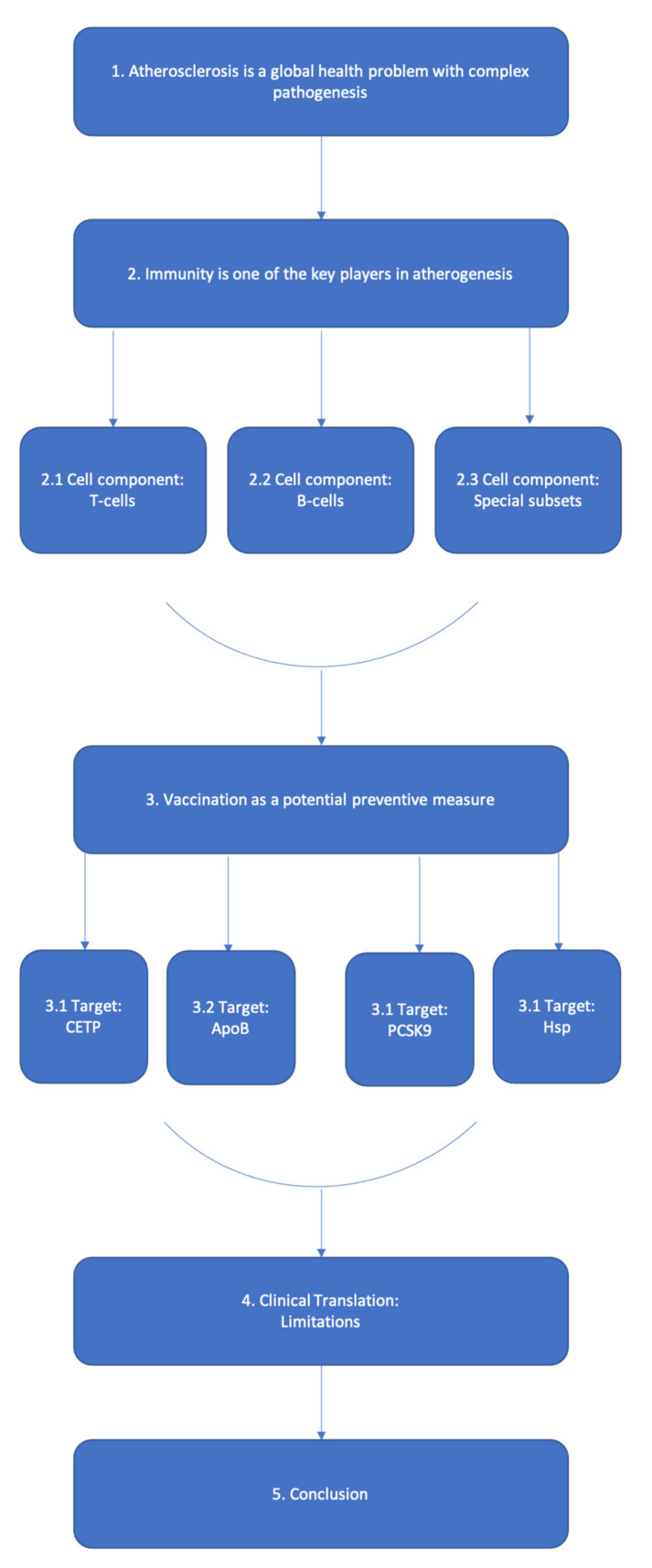
The scheme of the text.

The scheme of our review is depicted in Scheme 1.

Atherosclerosis can be considered the main enemy of modern medicine in every developed country. Vascular damage in atherosclerosis is triggered by unknown means, is self-sustaining and progresses with well-studied events. Having studied the factors that exacerbate the progression of atherosclerosis, it is easy to see that these factors are related to a so-called unhealthy lifestyle [1,2].

Atherosclerosis usually does not cause signs and symptoms until it severely narrows or totally blocks an artery. Atherosclerosis can lead to serious problems, including heart attack, stroke, or even death. Atherosclerosis can affect any artery in the body, including arteries in the heart, brain, arms, legs, pelvis, and kidneys. As a result, different diseases may develop based on which arteries are affected [3].

At the same time, pathologists have found the first stage of atherosclerosis (fatty streaks) in children who have died from causes other than atherosclerosis or obesity.

The first stage of atherosclerosis is characterized by the accumulation of LDL (low-density lipoprotein) and VLDL (very low-density lipoprotein) under the endothelium, which contributes to the development of endothelial damage and the launch of inflammation. Not all streaks become plaques, because the lipids may not accumulate in the region due to gradual removal by macrophages and good HDL (hence, they are “anti-atherogenic”) [4,5].

Next, monocytes arrive at the inflammation site, some of which become macrophages and begin to absorb the accumulated lipids. Over time, they become unable to exit back into the bloodstream and remain in the endothelium. Although some still exit into the bloodstream, as they do not interfere with the other macrophages that have grown due to their constant consumption of lipids. The remaining foam cells eventually die and shed all the stored fat back under the endothelium, forming the lipid core of a fully grown and solid plaque [6].

As the foam cells accumulate and the plaque grows, “helpers” begin to be drawn into the plaque not only from the blood, but also from the middle layer of the artery. Muscle cells gradually appear in the plaque, which eventually disintegrate by apoptosis. Initially the monocytes attend the site to manage the excess of lipoproteins, subsequently there are a huge number of macrophages, smooth muscle cells, and lipids, which attract monocytes: thus a vicious circle is formed, because the newly arrived cells do not naturally leave the site, but only aggravate the condition [7].

There is a theory that everything is tied to the relative charge of the tissues: the vascular endothelia, like platelets, have a positive charge, and different charges are repelled, so the blood flows without friction [8]. When the charge drops on the vessel wall, that is, when the endothelium is damaged, platelets begin to “stick”. To “save” the situation and close the gap, the body puts a patch of cholesterol dielectric.

Atherosclerosis leads to the two most common causes of death, covering 25%, or more than 14 million deaths annually worldwide, with a tenfold gap to HIV/AIDS or car accidents. In fact, if the act of pathoanatomical autopsy would write the cause not of death, but of the original condition, it would be atherosclerosis.

Vaccination is proved to be the best preventive measure for vast majority of diseases related to inflammation. However, the development of the applicable vaccine is often complicated with the intricacy of pathogenesis. Thus, it is still unknown, if vaccination in atherosclerosis is beneficial. The biological properties of the epitope of the vaccine are extremely important due to ability of the epitope to define the nature of vaccine-induced immunity. For example, live vaccines contain attenuated variants of pathogens, which do not lose the ability to activate immature dendritic cells and other antigen-presenting cells (APCs). However, subcellular- or subunit-based vaccines often lack this immunogenic potential. Therefore, vaccines of such origin are typically combined with adjuvants to increase and modulate the vaccine’s immunogenicity via longer lasting and more effective activation of immune cells.

## 2. Immunity in Atherogenesis

The role of immunity in the progression of atherosclerosis is mentioned by various authors [9]. The modern assessment of atherogenesis from the perspective of inflammation allows us to consider the kinetics of arterial wall cells, taking into account the expression of cytokines and intercellular cooperation: macrophage—T lymphocyte—smooth muscle cell [10]. Activated white blood cells and monocytes participate in a “metabolic explosion”, releasing active radicals produced by the reactions of lipid peroxidation. In this case, endotheliocyte damage occurs followed by the formation of an atherosclerotic plaque. The expression of proinflammatory cytokines and growth factors is accompanied by cell proliferation [11].

In atherosclerosis, several types of immunocompetent cells are involved in the inflammatory process, primarily monocytes, B and T lymphocytes, and possibly mast cells (mastocytes). In the process of atherosclerotic inflammation, the key role belongs to monocytes/macrophages [12]. The role of macrophages in immunity is extremely important—they provide phagocytosis; process and present antigens to T cells; and they secrete lysozyme, neutral proteases, acid hydrolases, arginase, and many complementary components including enzyme inhibitors (plasminogen anti-activator, a2-macroglobulin), transport proteins (transferrin, fibronectin, transbalamin II), nucleosides and cytokines (TNF-a, IL-1, IL-8, IL-12) [13]. IL-1 has many important functions: affecting the hypothalamus; causing fever; stimulating the release of neutrophils from the bone marrow; and activating lymphocytes and neutrophils. Macrophages are one of the tools of innate immunity. In addition, macrophages, along with B and T lymphocytes, are also involved in the acquired immune response, being an “additional” type of immune response cells. Macrophages are phagocytic cells whose function is to “swallow” immunogens and process them for presentation by T lymphocytes in a form suitable for the immune response [14]. T lymphocytes recognize an infected macrophage by the presence of a microbial antigen on its surface, which is in a complex with the major histocompatibility complex (MHC) class II glycoprotein, which in this case serves as a macrophage signal. As a result of the recognition, T cells secrete lymphokines that stimulate intracellular destruction of the pathogen by macrophages [15]. Thus, therapy aimed at correcting the monocyte–macrophage system is a priority in patients with an inflammatory cause of the disease, at all stages of the inflammatory process, and regardless of its location. To assess the possible effect of drug therapy on slowing the progression of the disease, it is important to study the dynamics of the levels of proinflammatory cytokines [16]. It should be noted that atherogenic classes of lipoproteins are potentially pro-inflammatory factors. This applies to triglyceride-rich lipoprotein-chylomicrons, very low-density lipoproteins (VLDL), and especially low-density lipoproteins. In contrast, high-density lipoproteins (HDL) are anti-inflammatory factors [17].

The observations of Hansson and co-authors [18], which showed that atherosclerotic lesions of the arteries contain CD3+ cells, initiated the study of the role of T lymphocytes in atherosclerosis. Since then, a large body of information has been produced showing that T lymphocytes are key immunoregulatory cells involved in atherosclerosis. The presence of B lymphocytes and plasma cells in atherosclerotic lesions has also been shown [19]. The adaptive response in atherosclerosis is represented by the cellular and humoral components of the response. Cellular immunity in atherogenesis involves T helper lymphocytes (CD4+), as well as cytotoxic T lymphocytes (CD8+), while the humoral response is carried out by B cells producing immunoglobulins [20]. The balance between cellular and humoral responses is regulated by CD4+ T lymphocytes. Populations of these cells can produce almost the entire spectrum of cytokines, but an effective response, cellular or humoral, can only be achieved by activating certain populations of these cells—highly polarized subtypes of T lymphocytes: TY- and Th2-cells. CD4+ lymphocytes are divided into several subpopulations, including TY and Th2. The terms “TH-cytokines” and “Th2-cytokines” reflect the origin of the cytokines secreted by these two populations of T helper cells [21]. Sometimes cytokines produced by the corresponding Th subtypes of cells are called type 1 and type 2 cytokines. Type 1 cytokines (or TY-cytokines) include interleukins IL-2, IL-12, IL-18, interferon-y( IFN-y), TNF-a; and type 2 cytokines (Th2-cytokines) include interleukins IL-4, IL-5, IL-6, IL-9, IL-10, IL-13, and transforming growth factor beta (TGF-P) [22].

### 2.1. T Cell Research in Atherosclerosis

The antigenic specificity of T cells is still unknown. In published studies of T cells in atherosclerosis, there is no information about this. There is no definition of antigen specificity, which is unexpected given that most of the functions of T cells are precisely dependent on the antigen. But determining the antigenic specificity of T cells in any other disease, not just atherosclerosis, is technically very difficult. Two methods may be used to search, isolate, and study antigen-specific T cells: tetramers and restimulation assays [23].

MHC class II tetramers loaded with an antigen peptide can bind to CD4+ T cells, and MHC class I tetramers loaded with an antigen peptide can bind to CD8+ T cells. Tetramers are recombinant proteins that must be specially developed for the antigenic peptide under consideration and for the specific MHC alleles expressed by the object of the study [24]. Each tetramer should be tested for non-specific binding to other white blood cells. To establish specificity, the tetramer should not bind to T cells in people with MHC mismatch. The tetramers should co-cluster with the T cell receptor (TCR) on individual cells. The number of cells identified by tetramers is usually small, in the order of ten cells per million. In atherosclerosis, there are few studies of tetramer-isolated T cells [25].

Restimulation assays can be performed with mixtures of antigenic peptides loaded on APCs. Normally, APCs are gained from the same person whose T cells are to be interrogated. Thus, their MHC alleles naturally correspond to the endogenous TCR repertoire. In the restimulation analysis, similar to tetramers, it is not known whether the synthetic peptides that trigger the T cell response are naturally represented peptides. T cell hybridomas were obtained with T cells from atherosclerotic LDL^–/–^mice [23]. The limitation of T cell hybrids is that they grow independently of external stimuli, which distorts the T cell phenotype. Thus, almost all CD4+ T cell hybridomas reported in atherosclerosis belong to T helper 1 (Th1), although many other T cells are found in atherosclerotic lesions in situ [26]. 

Due to these technical restrictions, the antigenic specificity of various subsets of T cells is unknown in nearly all studies of atherosclerosis. This lack of determination of the specificity of the T cell antigen may be one of the reasons for the significantly different effects of subsets of T cells in atherosclerosis reported in experimental studies, as a result of which the same subset of T cells may be proatherogenic when recognizing an antigen related to atherosclerosis, and potentially neutral or antiatherogenic when detecting an unrelated antigen. This lack of knowledge about antigen specificity limits the conclusions that can be drawn about the role of subsets of T cells in atherosclerosis [27].

Immunohistochemical studies, single-cell RNA sequencing (scRNA-seq), and mass cytometry (CyTOF) have shown that 25–38% of all white blood cells in mouse aortic atherosclerotic plaques and human atherosclerotic plaques are CD3+ T cells [28,29,30]. A study applying the innovative methodology of cellular indexing of transcriptomes and epitopes by sequencing (CITE-seq) showed that T cells make up the majority of immune cells in human atherosclerotic plaques obtained as a result of endarterectomy [29]. Nevertheless, flow cytometry, CyTOF, scRNA-seq, and CITE-seq require mechanical dissociation and enzymatic digestion of tissues to prepare single-cell suspensions. Enzymes used for tissue digestion can alter cell surface molecules by proteolytic cleavage, and cellular processes occurring during digestion and excretion can alter single-cell transcriptomes. In addition, isolation procedures can affect the ratio of different cell types and distort it. Because T cells are small, round, and mechanically strong, these cells may survive the isolation procedure better than macrophages or dendritic cells, which are large, branched, and brittle [31]. Analysis of gene expression signatures obtained using CITE-seq and scRNA-seq showed that human atherosclerotic plaques are enriched with T cells that exhibit cytotoxicity, activation, and depletion, while T cells in the blood have gene signatures associated with cytokine inhibition, RNA synthesis, and metabolic reprogramming [29]. Studies in humans and mice have shown that T cells preferentially populate atherosclerotic lesions with the enrichment of the fibrous membrane but are also found in the adventitia of older lesions [32].

The T cell population of atherosclerotic plaque is dominated by Th1-type CD4 T cells [33]. These cells have the capacity to produce proinflammatory and immune-stimulating cytokines, including TNF-α and interferon-γ. Th1 cells are powerful promotors of plaque inflammation by activating macrophages, endothelial cells, and smooth muscle cells [34]. In addition to Th1 cells, CD8 cells are enriched with plaques versus peripheral blood [29]. The less common T cell types in the plaque include Treg, Th17, and Th2. Natural killer T cells, a T cell type specialized for detection of certain lipids, can also be found in modest numbers in plaques. A recent single-cell proteomic and transcriptomic analysis showed that T cells of human plaques were more differentiated, activated, and in part, exhausted than those in peripheral blood [35]. Transcripts indicative of effector memory phenotypes and of adaptive immune activation were increased, whereas signals of regulatory immunity were reduced as compared to blood T cells. Importantly, symptomatic plaques were characterized by increased interferon-g, type I interferon, and TNF signaling in CD8 as well as CD4 T cells [36].

### 2.2. B Cells and the Development of Humoral Immunity

B cell differentiation is even more complex than that of T cells. Not only does it require antigen recognition and T cell assistance but also involves conversion of antigen receptors into secreted immunoglobulin antibodies. Their first round of activation leads to the production of IgM antibodies. Renewed encounters with antigens will, with the help of T cells, trigger a cascade of events. The antibody gene is reshuffled to produce smaller IgG (as well as IgA and IgE) antibodies, large amounts of these antibodies are secreted, and the cell proliferates to form clones of cells producing the same antibody [37]. If immune stimulation ceases, memory B cells will maintain the capacity for antibody production if the antigen appears again. Further antigenic stimulation, on the other hand, drives further differentiation of the B cells in the lymph nodes into plasma cells specialized in producing significant amounts of specific antibodies. The role of B cells in atherosclerosis is complex, with protective as well as disease-promoting activities [38].

### 2.3. T and B Cell Subsets with Distinctive Functions

In parallel with classical B cell differentiation, termed the B2 pathway, other B cells exist in which antibody genes do not reshuffle upon activation, and plasma cells do not form. Such cells are termed B1 cells, they produce IgM antibodies, and do not depend on T cell help. A significant proportion of B1 cells recognize phospholipids through germ-line encoded antibodies referred to as natural antibodies. This response is particularly important for protection against encapsulated pneumococci. It has also been implicated in atherosclerosis because several studies have shown that natural antibodies reduce disease development [39].

A group of scientists from Australia, studying the interaction of B cells and CD4 T cells, came to the following conclusions [40]. First, life-long deficiency of B cells reduces atherosclerosis. Reduced atherosclerosis in B cell deficient mice, is associated with decreased levels of immunoglobulin. B cell deficiency lowers lesion CD4 T cells and reduces the production of proinflammatory cytokines, such as IL-1β, TGF-β, MCP-1, M-CSF, and MIF. Second, MHC II and CD40 expressed by B cells are required for the interaction between B and T cells in atherogenesis. Lastly, B-cell specific expression of MHC II and CD40 is needed for both B and CD4 T cell effector functions.

## 3. Vaccination

Could it be possible to prevent this illness by vaccination? Göran K. Hansson, MD, PhD in his article explores whether it is possible, guided by published research [41].

**Figure 1:**
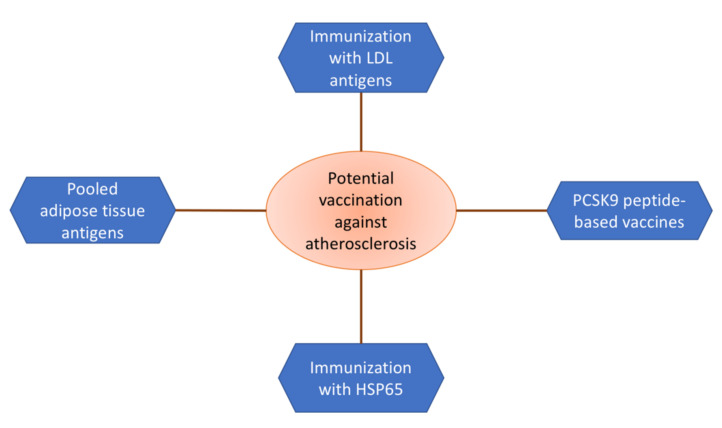
Variety of approaches to the development of an atherosclerosis vaccine.

Clinical trials based on experimental models show that diseases such as rheumatoid arthritis and multiple sclerosis may be prevented by vaccination. Both of these diseases are inflammatory diseases with complex autoimmune processes. Despite the fact that atherosclerosis cannot be considered a “fully fledged” autoimmune disease, it has much in common with the above-mentioned ailments [42]. Accordingly, it is possible to prevent atherosclerotic cardiovascular diseases using the same approaches. The most promising are summarized in Figure 1.

Two gene-targeted murine models of atherosclerosis, the apolipoprotein E knockout and the LDL-receptor knockout mice, possess substantiated to live workable for this research [42,43,44].

Various evidence has demonstrated that CD4 T cells may have a negative effect on atherogenesis. In a case of transfer of these cells from immunocompetent to immunodeficient mice with apolipoprotein E knockout, the disease grew instantly [43]. The CD4 cell is the most common T cell in atherosclerotic damage and shows responsiveness to putative antigens [44]. CD4 T cells can be classified into two dissimilar subgroups that balance each other [45]. The T helper cell (Th1) is a prevalent type of CD4 T cell that induces macrophage activation and increases inflammatory Th1 cells mostly by secreting interferon, an indispensable pro-inflammatory cytokine that is created in human atherosclerotic lesions and hastens atherosclerosis in mice [46]. In contrast, the Th2 cell, inhibits inflammation and decreases macrophage activity. Various other cytokines may also contribute to this result including interleukin-4, interleukin-10 (IL-10), and transforming growth factor. Oddly, Th2 activation may be hastened by oral or nasal injection of antigens [43,47]. In experimental autoimmune conditions, like experimental autoimmune encephalomyelitis and collagen-induced arthritis, the introduction of myelin and cartilage proteins, one by one, has been used to efficaciously impede the progression of the ailment [48]. Mucosal vaccination is presently being trialed on human analogues for MS, and rheumatoid arthritis [49].

### 3.1. Targeting CETP

Other vaccination studies related to atherosclerosis include vaccination against CETP (cholesterol ester transfer protein) to increase HDL (high-density lipoprotein) cholesterol, angiotensin II to decrease blood pressure, and oral administration of pooled adipose tissue antigens in overweight patients to induce a more favorable lipid profile [50,51,52,53]. The clinical development of these vaccines was later discontinued, with the exception being the adipose tissue antigen vaccine which is still under investigation. Another interesting, alternative approach to achieve atheroprotective immune modulation was the LILACS trial (Low-Dose Interleukin-2 in Patients with Stable Ischemic Heart Disease and Acute Coronary Syndromes) that aimed to study the effect of increasing Treg cells by treatment with low dose IL-2 [50,51].

### 3.2. Targeting ApoB

Atherosclerosis vaccines that are constructed on the principle of modulating autoimmune responses against LDL-associated antigens are yet to be tested in clinical trials. Based on the experimental evidence, a significant mode of action of such vaccines would be to suppress Th1-dependent proinflammatory immune responses against antigens formed in modified LDL particles. This suppression would be mediated by antigen-specific Tregs that become activated when exposed to their cognate antigen by macrophages and other antigen-presenting cells in the atherosclerotic plaques [52]. Theoretically, these antigen-specific Tregs would not only suppress the activity of Th1 T cells with corresponding antigen specificity but could also inhibit plaque inflammation through the release of anti-inflammatory cytokines, such as IL-10 and TGF-β. This mode of action has the advantage of having a lower risk of the adverse side effects associated with systemic anti-inflammatory therapy. Thus, it would not be expected to cause the small increase in frequency of fatal infections of the kind observed in the CANTOS (Canakinumab Anti-Inflammatory Thrombosis Outcome Study) trial [50]. Mucosal administration of apoB peptides, as well as subcutaneous injection of apoB peptides with or without aluminum-based adjuvants, have all been shown to inhibit atherosclerosis in experimental models through the activation of Tregs [53,54,55]. Data on animal studies targeting various antigens are summarized in Table 1.

In addition to regulatory immunity, atherosclerosis vaccines could also induce disease protection by inducing production of high-affinity antibodies to LDL [54]. Such antibodies facilitate clearance of LDL from the circulation [52]. They may also neutralize LDL particles in the extracellular space, thus preventing their ligation of pattern recognition receptors, in turn reducing intracellular cholesterol accumulation and also inhibiting induction of proinflammatory signals in macrophages [3].

The European Union-sponsored program Vaccination in Atherosclerosis (http://www.viavaccine.com, accessed on 20 January 2022) brought together a group of academic institutions, small and medium-sized enterprises, and industrial partners with the goal of bring an LDL-based atherosclerosis vaccine into phase I clinical testing [52]. The Vaccination in Atherosclerosis group developed vaccine formulations based on different apoB peptide and phospholipid antigens, investigated how clinically approved adjuvants could be optimized for binding of apoB peptides, compared different routes of administration in experimental animal models, performed mechanistic studies to more exactly characterize the mode of action, evaluated candidate biomarkers to monitor the effect of vaccination in ex vivo lymphoid models, and studied the association of these biomarkers with the risk of cardiovascular events in prospective population cohorts. Vaccination in Atherosclerosis investigators also had a continuous dialogue with regulators and pharmaceutical companies about safety requirements and other issues that needed to be resolved before a vaccine clinical program could be initiated [60,61].

At the end of the five-year program, the Vaccination in Atherosclerosis group concluded that too many safety issues remained unsolved to allow the start of phase 1 testing of an atherosclerosis vaccine based on apoB peptides. It was recommended that clinical programs instead, should focus on apoB-specific antibodies. If such antibodies could be proved to lower cardiovascular risk in clinical trials, it would open the path for the development of vaccines that induce the expression of antibodies with the same specificity. Vaccines that induced LDL tolerance specifically in atherosclerotic plaques were still seen as the most optimal therapeutic alternative in the long-term, but the clinical testing of such vaccines would require a better understanding of the mode of action, how to monitor the response to vaccination, their effectiveness in advanced stages of atherosclerosis, as well as a better understanding of potential safety issues [62].

### 3.3. PCSK9

Treatment with antibodies against PCSK9 effectively lowers LDL cholesterol and has been shown to further reduce cardiovascular risk when used in combination with statins [63]. Human mAbs against PCSK9 (alirocumab and evolocumab) have already been approved by the European Commission (EC) and the US Food and Drug Administration (FDA) as adjunct therapy for hypercholesterolemia.

However, the high costs of treatment limit the use of PCSK9 antibodies in wider patient groups. Accordingly, the possibility of inducing similar antibodies by vaccination has gained attention as a more cost-effective approach. 

The use of such vaccines has been evaluated in animal models. For example, nanoliposomal construction L-IFPTA+ was found to successfully induce a humoral immune response against PCSK9 in BALB/c mice. The production of antiPCSK9 antibodies in BALB/c mice in response to L-IFPTA+ vaccine administration was long lasting and immunogenically safe [56]. The safety, PCSK9 antibody response, and LDL-lowering capacity of two PCSK9 peptide-based vaccines have been tested in a phase I trial, but the results have not yet been published [63,64].

### 3.4. Heat Shock Proteins

There are studies that have shown a positive effect of immunization with LDL antigens [44,65,66], which demonstrate the protective effect of nasal and oral immunization with another antigen, heat shock protein 65 (HSP65) [43].

Decreased lesion size, as well as increased splenic Treg numbers, increased interleukin (IL)-10 mRNA levels in the aorta, increased levels of anti-mbHSP65, and anti-mouse HSP60 antibodies pointing to a pro-eukaryotic HSP60 humoral cross reaction, not curtailed by oral tolerization, were observed in ApoE^–/–^ mice immunized with mbHSP65 protein or peptides, given mbHSP65 orally and then kept either on chow or a high cholesterol diet [57]. In 2016, Zhong et al. presented the results of their investigations, in which a significant, 33·6% reduction in plaque size at the aortic root in the early stages of atherosclerosis was observed after immunization. The vaccine contained HSP60 and was administrated by nasal route [59].

In another study, interesting results were obtained in ApoB(tm2Sgy)/Ldlr(tm1Her/J) knockout mice. The efficacy of atherosclerosis prevention was assessed for peptides derived from apolipoprotein B100 (ApoB), heat shock protein 60 (HSP60), and complement cascade (peptide A). Both ApoB and HSP60 appeared to reduce early atherosclerotic lesions in mice by 32.1 and 33.5%, respectively, while the reduction with peptide A was 5.7% [58].

## 4. Clinical Translation

**Figure 2:**
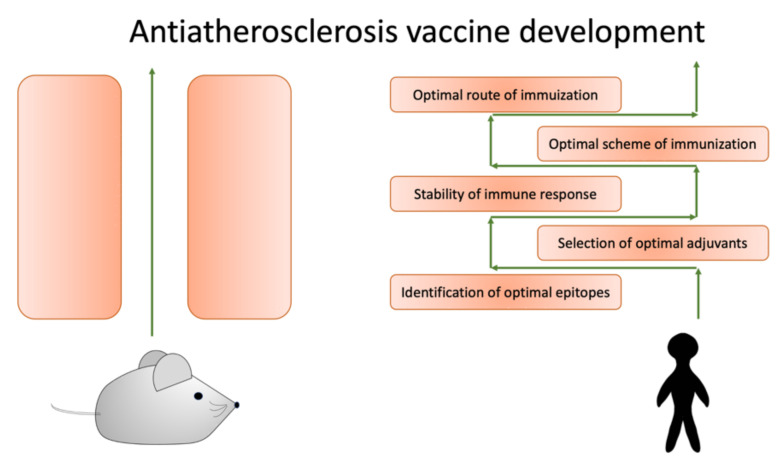
To successfully translate data obtained from model animals, we need to overcome several obstacles.

The translation of the obtained preclinical evidence of potentially beneficial anti-atherosclerotic vaccines to clinical practice remains challenging. There are several crucial limitations to overcome before these strategies can be effectively used in clinical practice. These obstacles are summarized in Figure 2.

One of the main problems is the identification of optimal epitopes. Vaccines activating the CD4^+^ T cell response require the exact allele of the HLA complex. It would be ideal to create a version of an ApoB protein that has the same, close to 100%, capacity to bind to all HLA types and alleles. However, not all ApoB peptides bind to all HLA types with similar affinity [67]. A significant investigation in this field was performed by Gistera et al. in 2018. The authors used HuBL mice to identify the epitopes from human apolipoprotein B100 that triggered activation of T-cells. Two epitopes were evaluated, the action of which led to protection from atherosclerosis after vaccine administration [68].

A list of 30 ApoB peptides that bound to broadly different HLA types was generated by a computer algorithm-based prediction and in vitro affinity measurement. The list was presented in the paper by Wolf et al., with the results of the successful induction of in vitro responses of T cells isolated from healthy volunteers and patients with coronary artery disease by the peptide pool [69].

Another approach provided in clinical trials of cancer vaccines is the Ii-Key hybrid technology. This technology presents the a fragment of the Ii protein (or MHC class II-associated invariant chain), which physiologically binds to MHC-II molecules. Ii-key binds to all HLA-types with high affinity. However, this technology has not been tested in atherosclerosis as yet [70].

Another problem to consider is the design and selection of effective and clinically applicable adjuvants. This essential vaccine component must meet several criteria, which include the antigen used, the desired immune response, and administration route. However, the widespread adjuvants, such as Freund’s adjuvant (CFA or IFA), aluminum, and cholera toxin B subunit, are not good variants because of potential side effects [71].

A good result was demonstrated for Addavax, a squalene-based adjuvant similar to MF59, which has been approved as an influenza vaccine in several countries including the USA. The vaccination with Addavax stimulated IL-10 production in restimulated cells but did not induce an antigen-specific antibody response. An exact mechanism of protection is not completely understood, but Addavax has emerged as a promising candidate for clinical translation of a vaccine against atherosclerosis [60].

Another strategy for overcoming the adjuvant selection problem is to develop adjuvant-free vaccines. Such an approach was demonstrated in work by Herbin et al., who performed adjuvant-free continuous delivery of a low-dose ApoB–peptide mix (p210, p240, and MDA-p210) [72].

One more approach consisted of liposomes, which were used as capsules for ApoB peptides. This drug-delivery system is a promising developing strategy and has implications for various disease treatment strategies [73].

The most intriguing limitation is the stability of the atheroprotective immune response. Immunization with MHC-II-restricted ApoB peptides acts through the expansion of ApoB^+^ T_regs_. Yet, the development of atherosclerosis in *Apoe*^–/–^ mice is accompanied by a conversion of T_regs_ into proatherogenic T_FH_ cells or dysfunctional IFN-γ-producing T_H_1/T_reg_ cells with reduced suppressive capacities. Recent evidence suggests that ApoB^+^ T cells are especially prone to switching their phenotypes under hypercholesterolemic conditions. An exact mechanism for determining the deleterious T cell conversion in atherosclerosis remains unclear. Clinical translation of a T_reg_-based vaccine strategy requires a fundamental understanding of the switch underlying the mechanism. Vaccination could cause serious harm when T_regs_ are induced, as they may subsequently convert into disease-promoting effector T cells [69].

The development of an optimal scheme of immunization is another obstacle. Studies on murine models have revealed that the schedule of vaccination is of particular importance. A usual protocol for vaccination follows a preventive approach aiming to induce atheroprotective immunity before disease onset. However, the development of tertiary prevention (for the treatment of patients who have already developed atherosclerosis) is also an intriguing idea. However, considering the impact of the T_reg_ switch in atherosclerosis, immunization of patients with established disease must be carefully assessed [74].

## 5. Conclusions

In atherosclerosis, immune responses against a subject’s own antigens play an important role, but atherosclerosis cannot be classified as an autoimmune disease in the classical sense. Much is still unknown about the role of the immune system in atherosclerosis, but based on current knowledge, data, and trials, it appears that protective innate and adaptive immune responses, including antibodies that help clear potentially toxic waste products, may protect against atherosclerosis.

The data presented in this review indicate that inflammation is one of the leading components of the pathogenesis of atherosclerosis and affects the rate of its progression, the nature of lipid metabolism disorders, and the severity of clinical manifestations.

Thus, atherogenesis is closely related to the protective response to inflammation, in which an attempt to localize the inflammatory zone leads to an excessive fibroproliferative cellular response, often with a narrowing of the vessel lumen. The sequence of biological events within the vessel wall that occur during such a protective response includes the penetration of inflammatory cells into the subendothelial space, the accumulation of lipids, the activation and degranulation of platelets, and the migration and proliferation of stromal cells synthesizing the extracellular matrix. In the future, work should continue to identify the relationship between hypercholesterolemia, immunocompetent cells, and the development of atherosclerotic lesions, which should lead to the development of new ways to control the immune components of atherogenesis.

Despite all the difficulties that arise when trying to introduce promising vaccines against atherosclerosis into clinical use, these problems may be overcome in the future. At the same time, it will be important to identify patients with residual risk on the current treatments, who would benefit from immunomodulatory therapy. Based on current knowledge, it is likely that patients with autoimmune diseases, which are known to be associated with increased cardiovascular risk and for which traditional preventive treatments are ineffective, represent one of these groups.

## Figures and Tables

**Table 1:** Summary of experimental studies targeting various antigens.

Target	Model	Result	Reference
apoB	hypercholesterolemic huB100(tg) × Ldlr^–/–^ mice	Inhibition of atherosclerosis through the activation of Tregs	[53]
apoB	ApoE^–/–^	Inhibition of atherosclerosis through the activation of Tregs	[55]
PCSK9	BALB/c mice	Induction of humoral immune response against PCSK9	[56]
mbHSP65	ApoE^–/–^ mice	Lesion size decrease	[57]
HSP60, apoB	ApoB(tm2Sgy)/Ldlr(tm1Her/J) mice	Induced T-cell proliferation and expansion of regulatory T cells with interleukin-10 and transforming growth factor-β secretion	[58]
HSP60	Six-week-old male ApoE^–/–^ mice from a C57BL/6J background	Significant 33·6% reduction in plaque size at the aortic root in the early stages of atherosclerosis	[59]

## Data Availability

Not applicable.
